# Electroacupuncture alleviates the relapse of pain-related aversive memory by activating KOR and inhibiting GABAergic neurons in the insular cortex

**DOI:** 10.1093/cercor/bhad321

**Published:** 2023-09-07

**Authors:** Siqi Xiao, Haiju Sun, Yichen Zhu, Zui Shen, Xixiao Zhu, Ping-an Yao, Yifang Wang, Chi Zhang, Wei Yu, Zemin Wu, Jing Sun, Chi Xu, Junying Du, Xiaofen He, Jianqiao Fang, Xiaomei Shao

**Affiliations:** Key Laboratory of Acupuncture and Neurology of Zhejiang Province, Department of Neurobiology and Acupuncture Research, The Third Clinical Medical College, Zhejiang Chinese Medical University, Hangzhou 310053, China; Key Laboratory of Acupuncture and Neurology of Zhejiang Province, Department of Neurobiology and Acupuncture Research, The Third Clinical Medical College, Zhejiang Chinese Medical University, Hangzhou 310053, China; Key Laboratory of Acupuncture and Neurology of Zhejiang Province, Department of Neurobiology and Acupuncture Research, The Third Clinical Medical College, Zhejiang Chinese Medical University, Hangzhou 310053, China; Key Laboratory of Acupuncture and Neurology of Zhejiang Province, Department of Neurobiology and Acupuncture Research, The Third Clinical Medical College, Zhejiang Chinese Medical University, Hangzhou 310053, China; Key Laboratory of Acupuncture and Neurology of Zhejiang Province, Department of Neurobiology and Acupuncture Research, The Third Clinical Medical College, Zhejiang Chinese Medical University, Hangzhou 310053, China; Key Laboratory of Acupuncture and Neurology of Zhejiang Province, Department of Neurobiology and Acupuncture Research, The Third Clinical Medical College, Zhejiang Chinese Medical University, Hangzhou 310053, China; Key Laboratory of Acupuncture and Neurology of Zhejiang Province, Department of Neurobiology and Acupuncture Research, The Third Clinical Medical College, Zhejiang Chinese Medical University, Hangzhou 310053, China; Key Laboratory of Acupuncture and Neurology of Zhejiang Province, Department of Neurobiology and Acupuncture Research, The Third Clinical Medical College, Zhejiang Chinese Medical University, Hangzhou 310053, China; Key Laboratory of Acupuncture and Neurology of Zhejiang Province, Department of Neurobiology and Acupuncture Research, The Third Clinical Medical College, Zhejiang Chinese Medical University, Hangzhou 310053, China; Department of Acupuncture and Moxibustion, The First Affiliated Hospital of Zhejiang Chinese Medical University, Hangzhou 310060, China; Key Laboratory of Acupuncture and Neurology of Zhejiang Province, Department of Neurobiology and Acupuncture Research, The Third Clinical Medical College, Zhejiang Chinese Medical University, Hangzhou 310053, China; Key Laboratory of Acupuncture and Neurology of Zhejiang Province, Department of Neurobiology and Acupuncture Research, The Third Clinical Medical College, Zhejiang Chinese Medical University, Hangzhou 310053, China; Key Laboratory of Acupuncture and Neurology of Zhejiang Province, Department of Neurobiology and Acupuncture Research, The Third Clinical Medical College, Zhejiang Chinese Medical University, Hangzhou 310053, China; Key Laboratory of Acupuncture and Neurology of Zhejiang Province, Department of Neurobiology and Acupuncture Research, The Third Clinical Medical College, Zhejiang Chinese Medical University, Hangzhou 310053, China; Key Laboratory of Acupuncture and Neurology of Zhejiang Province, Department of Neurobiology and Acupuncture Research, The Third Clinical Medical College, Zhejiang Chinese Medical University, Hangzhou 310053, China; Key Laboratory of Acupuncture and Neurology of Zhejiang Province, Department of Neurobiology and Acupuncture Research, The Third Clinical Medical College, Zhejiang Chinese Medical University, Hangzhou 310053, China

**Keywords:** GABAergic neurons, insular cortex, kappa opioid receptor, pain-related aversive memory behaviors, electroacupuncture

## Abstract

Pain-related aversive memory is common in chronic pain patients. Electroacupuncture has been demonstrated to block pain-related aversive memory. The insular cortex is a key region closely related to aversive behaviors. In our study, a potential mechanism underlying the effect of electroacupuncture treatment on pain-related aversive memory behaviors relative to the insular cortex was investigated. Our study used the chemogenetic method, pharmacological method, electroacupuncture intervention, and behavioral detection. Our study showed that both inhibition of gamma-aminobutyric acidergic neurons and activation of the kappa opioid receptor in the insular cortex blocked the pain-related aversive memory behaviors induced by 2 crossover injections of carrageenan in mice; conversely, both the activation of gamma-aminobutyric acidergic neurons and inhibition of kappa opioid receptor in the insular cortex play similar roles in inducing pain-related aversive memory behaviors following 2 crossover injections of carrageenan. In addition, activation of gamma-aminobutyric acidergic neurons in the insular cortex reversed the effect of kappa opioid receptor activation in the insular cortex. Moreover, electroacupuncture effectively blocked pain-related aversive memory behaviors in model mice, which was reversed by both activation of gamma-aminobutyric acidergic neurons and inhibition of kappa opioid receptor in the insular cortex. The effect of electroacupuncture on blocking pain-related aversive memory behaviors may be related to the activation of the kappa opioid receptor and inhibition of gamma-aminobutyric acidergic neurons in the insular cortex.

## Introduction

Pain is a multidimensional experience composed of sensory-discriminative, affective-motivational, and cognitive-evaluative components. Pain-related aversiveness is an important negative emotion experienced by patients with chronic pain and causes a considerable amount of suffering (Cameron and Schoenfeld 2018). Additionally, it is usually the first symptom to manifest, emerging even earlier than anxiety and depression (Mercer Lindsay et al. 2021). A previous study reported that unpredictable aversive experiences lead to changes in depression- and anxiety-related behaviors (Cameron and Schoenfeld 2018), which makes treating chronic pain more difficult. Fields (1999) described the unique feature of pain aversiveness as a quality of “algosity”; when a person is in pain, they first feel aversive. Additionally, this pain-related aversive memory guides the mind and influences the person’s actions in response to noxious stimulation (Cahana and Jones 2007; Waisman et al. 2022), which may be key to the development of chronic pain refractoriness (Apkarian et al. 2009; Yi and Zhang 2011; Shao et al. 2016). In clinical trials, many patients with chronic pain experience aversive memory, such as phantom limb pain (Kaur and Guan 2018; Urits et al. 2019) or mirror image pain (Hu et al. 2021). Pain-related aversive memory seriously affects the quality of life of patients. Therefore, eliminating these pain-related aversive memory behaviors is beneficial for curing chronic pain. However, the mechanism underlying the association between chronic pain and pain-related aversive memory behaviors is still unknown.

Previous studies have shown that the insular cortex (IC) is an important brain region for the integration of pain sensation, emotion, reward, cognition, and memory information (Bermudez-Rattoni 2014; Rodríguez-García and Miranda 2016; Shi et al. 2020; Cabeza et al. 2021; Zhang et al. 2022). Research by Hadamitzky et al. (2015) revealed that the extinction of conditional taste aversion is related to the activity of the IC. Additionally, another study showed that the expression of FOS-LI neurons in the IC increased when rats showed conditioned place aversion (CPA) induced by formalin (Lei et al. 2004). Studies have indicated that direct electrical stimulation of the macaque insular region induces aversion (Caruana et al. 2011). Recent studies have also demonstrated that damaging the anterior insular cortex (aIC) prevents the development of CPA in the context of morphine withdrawal (Wang et al. 2016). Thus, we infer that the IC may be a key region in the formation of pain-related aversive memory behaviors.

The IC consists of both gamma-aminobutyric acidergic neurons (GABAergic neurons; Sun et al. 2020a) and glutamatergic neurons (Shillinglaw et al. 2018). GABAergic neurons in the IC potently control network excitability and play an important role in the inhibitory mediation of reward circuits (Sun et al. 2020a). Decreasing GABAergic synaptic transmission in layer 5 pyramidal neurons of the mouse insular cortex causes impairments in long-term depression and conditioned taste aversion memory (Toyoda et al. 2020). Enhancement of GABAergic synaptic transmission facilitates synaptic depression in layer 5 pyramidal neurons of the IC (Toyoda 2018). Furthermore, the current study suggests that GABAergic neurons in the IC are closely involved in morphine-induced conditioned place preference reconsolidation. Postretrieval excitation of GABAergic neurons in the IC had a long-lasting morphine-induced conditioned place preference suppression effect (Sun et al. 2020a). Another study found that a proportion of these GABAergic neurons were colocalized with the kappa opioid receptor (KOR; Tongjaroenbuangam et al. 2006). Pretreatment with a KOR antagonist or KOR-derived designer receptors exclusively activated by designer drug (DREADD) selectively inhibited the excitation of layer 5 GABAergic neurons in the IC (Pina et al. 2020). On the other hand, the KOR agonist had little effect on cortical excitation in an acute pain model (Yokota et al. 2016). However, it is unclear whether GABAergic neuron and KOR activity in the IC changes during pain-related aversive memory behavior relapse.

Electroacupuncture (EA), which is known for its substantial efficacy and lack of obvious side effects, has been demonstrated to cure pain and many emotional disorders (Li et al. 2014; Shen et al. 2020; Liao and Lin 2021). Recent studies have revealed that EA can inhibit GABAergic neurons in the ventrolateral periaqueductal gray, exerting significant antinociceptive effects in pain models (Zhu et al. 2019). Additionally, EA has been shown to relieve aversive behaviors induced by pain memory by downregulating theta power (Shen et al. 2019). However, whether EA relieves pain-related aversive memory behaviors induced by GABAergic neurons and KOR activity in the IC remains unclear.

Here, we used chemogenetic and pharmacological methods combined with CPA and mechanical paw withdrawal thresholds (PWTs) to demonstrate whether EA could block pain-related aversive memory behaviors induced by 2 crossover injections of carrageenan (Carr) through the activity of GABAergic neurons and KOR in the IC.

## Materials and methods

### Animals

Male C57BL/6 mice, aged 8–10 weeks, were utilized in all experiments. The mice were procured from the Laboratory Animal Center of Zhejiang Chinese Medical University, which has received official approval from the Association for Assessment and Accreditation of Laboratory Animal Care. The mice were distributed randomly into different groups, with 4 mice per cage furnished with corn cob padding. The mice were housed in a comfortable, stable living environment complete with ventilation, air filtration systems, temperature control (23–25°C), and a 12-h light–dark cycle, with food and water readily accessible.

### Animal model

A pain memory model was established as previously described by 2 crossover injections of Carr (Kissin et al. 2006). The model mice received 2 crossover injections of 0.5% Carr, with the first injection given on day 0 in the left hind paw and the second injection given on day 11 in the right hind paw. The dose for both injections was 25 μL. Mice in the control group were injected with 25 μL of normal saline (NS) on the same days and at the same locations.

### Virus construction and infection

The mice were anesthetized by intraperitoneal (i.p.) injection with 0.3% pentobarbital sodium and then fixed on a stereotaxic frame (RWD, 68025, Shenzhen, China) under a heating pad (RWD, 69000, Shenzhen, China) to maintain a constant temperature of 36°C. According to Paxinos and Franklin’s The Mouse Brain in Stereotaxic Coordinates (Fifth Edition), we confirmed the accurate location for injecting the insular cortex (relative to bregma: AP: +1.65 mm and ML: ±2.83 mm; DV: –2.55 mm from the pia mater).

A total of 100 nL of virus solution (AAV2/9-VGAT1-hM3Dq/hM4Di-mCherry-WPRE-pA or AAV2/9-VGAT1-mCherry-WPRE-pA; PT-489/PT-488/PT-0325, Wuhan BrainVTA Scientific and Technical Corporation) was unilaterally or bilaterally infused into the IC of the mice by an infusion pump (WPI, UMC4, Sarasota, FL, United States) at a rate of 60 nL/min. The needle was left in place for 8 min at the end of the infusion to prevent virus overflow. For the experiments involving the chemogenetic activation/inhibition of GABAergic neurons in the IC, CNO (2 mg/kg, intraperitoneally) was administered on days 11–12.

### Cannula implantation and microinjection

Under i.p. anesthesia with 0.3% pentobarbital sodium, the mice were fixed on a stereotaxic frame. Twenty-six-gage guide cannulae were bilaterally implanted into the IC (relative to bregma: AP: +1.55 mm and ML: ±2.80 mm; DV: –2.45 mm) and anchored to the skull with dental cement and stainless steel screws. U50488/norBNI/vehicle were given by a 10 mL microsyringe mounted on a microinfusion pump at a rate of 200 nL/min. After injection, the mice were given an additional 2 min for drug diffusion.

### Von Frey filament test

We used von Frey filaments to measure the mechanical PWTs on the bilateral hind paw of each mouse. The mice were individually placed in a plexiglass chamber on a wire-mesh platform to facilitate detection. After they had adapted to the chamber for ~30 min, filament probes were inserted onto their hind paws, and the pressure was gradually increased. If the mice suddenly retracted their paws, licked their claws, or flinched, the response was regarded as a positive mechanical withdrawal response. We defined 3 positive responses out of 5 stimuli as the PWT, with an interval between each measurement longer than 1 min. The PWTs were measured on different days, including baseline, days 0, 1, 3, 5, 7, 11, and 12.

### CPA test

CPA was performed as documented earlier. The shuttle box was composed of 2 opaque acrylic compartments that measured 30 × 30 × 30 cm. They were positioned in parallel and separated by a divide that had a square door (10 cm per side), allowing the mice free access to either conditioning chamber. Each compartment was labeled with either a horizontal or a vertical stripe to ensure visual distinction. The experiment consisted of 3 periods: preconditioning, conditioning, and postconditioning (3 tests). During the preconditioning phase, the mice were moved into the shuttle box and allowed to move freely for 10 min. The time spent in each compartment was recorded and used as a baseline (mice that spent more than 70% of the total time in either chamber were excluded from the experiment). The conditioning period consisted of 4 days (days −1 to 2). On day −1, the mice were confined to the nonconditioned compartment. On day 0, the mice received an injection of either 0.5% Carr or NS solution in the left hind paw. Subsequently, the mice were confined to the conditioning chamber and subjected to plantar stimulation using a 1 g von Frey filament for 30 min each day until day 2. On days 3, 10, and 12, postconditioning assessment was conducted under the same preconditioning conditions. On day 11, the mice were administered an injection of 0.5% Carr or NS solution accordingly in the right hind paw. The time spent by each mouse in the 2 compartments was recorded to compute the CPA score (time spent in the conditioning chamber during postconditioning − time spent in the conditioning chamber during preconditioning) to evaluate Carr-induced aversive memory behaviors.

### EA treatment

The mice were subjected to EA treatment on days 11 and 12. The bilateral hind limb acupoints “Zusanli” (ST36) and “Sanyinjiao” (SP6) were carefully selected for stimulation after immobilizing the mice. We inserted acupuncture needles measuring 0.16 × 7 mm directly into the acupoints, located 5 mm from the needle tip. The needles were then connected to a HANS-200A acupoint nerve stimulator. The stimulation parameters were set to 100 Hz for frequency and 0.3 mA for intensity for a total duration of 30 min. Other mice were subjected to the same immobilization treatment but did not receive EA stimulation.

### Immunohistochemistry

The mice were intraperitoneally anesthetized using 0.3% sodium pentobarbital solution. Next, they were perfused with 0.9% saline and 4% paraformaldehyde at a fixed rate. The brains were extracted and stored in 4% paraformaldehyde at 4°C overnight and then dehydrated using a 15 and 30% sucrose solution until they sank. Coronal sections, 20 μm in thickness, were cut by a cryostat frozen microtome (Thermo Fisher Scientific, NX50, United States). For immunofluorescence (IF), the sections were warmed to 37°C for 1 h and then washed with PBST 5 times at 10-min intervals. Subsequently, the brain sections were incubated in 10% donkey blocking buffer at 37°C for 1 h and then with primary antibodies, including anti-c-Fos (1:500, rabbit, ab190289, Abcam) or rabbit anti-GABA (1:500, rabbit, GTX125988, GeneTex), at 4°C for 24 h. The sections were then rewarmed to 37°C for 1 h, washed 5 times with PBST, and finally incubated with corresponding fluorophore-conjugated secondary antibodies (1:800) at 37°C for 1 h. They were later washed an additional 5 times with PSBT and incubated with 4,6-diamidino-2-phenylindole at the final stage. To visualize the fluorescence signals, a digital pathological section scanner was utilized.

### Statistical analysis

The statistical analysis in this study utilized a range of methods. Simple comparisons of data such as c-Fos expression were performed using Student’s *t*-test. ANOVA and post hoc analyses (1-way and 2-way) were utilized to analyze data from groups with multiple comparisons. Tukey’s post hoc test was used to evaluate the PWTs, and the LSD post hoc test was performed after 1-way ANOVA for the CPA test results and number of GABAergic neurons. Statistical significance is indicated in the results as ^*^P < 0.05, ^*^^*^P < 0.01, ^*^^*^^*^P < 0.001, and NS. All data are expressed as the mean ± SEM.

## Results

### Pain-related aversive memory behavior relapse is induced by 2 crossover injections of Carr

**Fig. 1:**
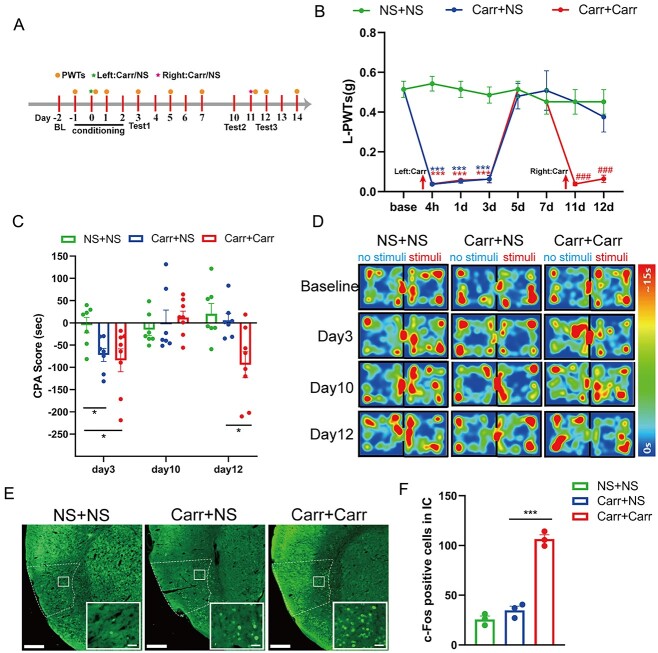
Two crossover injections of Carr established a pain memory model and induced pain-related aversive memory behaviors. A) Timeline for Carr injections and CPA testing. B) L-PWTs hindpaw in each group after model establishment (2-way ANOVA, ^*^^*^^*^P < 0.001, ^*^^*^^*^compared with the NS + NS group. ^###^*P* < 0.001, ^###^compared with the Carr + NS group. *N* = 7 mice). C) Carr injection induced a reduction in CPA scores (2-way ANOVA, ^*^P < 0.05, *n* = 7/8 mice). D) Heatmap of each group for several CPA tests. E) Sample images of c-Fos-positive cells in the IC in each group (scale bars: 200/20 μm). F) Number of c-Fos-positive cells in the IC between each group (1-way ANOVA, ^*^^*^^*^P < 0.001).

First, we successfully replicated the pain memory model with 2 crossover injections of Carr (Fig. 1A and B). We established a model of pain memory by injecting 0.5% Carr into the left hind paw on day 0 and the same dose of Carr into the right hind paw on day 11, and the model control was made by injecting 0.5% Carr into the left hind paw on day 0 and the same dose of NS into the right hind paw on day 11. We found that before the first Carr injection, there was no significant difference in the paw withdrawal thresholds in the left (L-PWTs) in each group. After the first injection, the L-PWTs of the Carr + NS group and Carr + Carr group were significantly lower at 4 h, days 3 and 5 than those of the NS + NS group (*P <* 0.001). Before the second injection, we allowed the L-PWTs of the mice in the Carr + NS group and Carr + Carr group to recover. On days 1 (day 11) and 2 (day 12) after the second injection, the L-PWTs in the Carr + Carr group were significantly lower than those in the Carr + NS group (*P* < 0.001; Fig. 1B). The results indicated that the pain memory model was successfully prepared. To verify whether pain memory can produce pain-related aversive memory behaviors, we conducted CPA testing at the corresponding times (Fig. 1A). The results showed that after the first injection, the CPA scores of the Carr + NS group and Carr + Carr group were significantly higher than those of the NS + NS group (*P* < 0.05), indicating that acute pain could induce pain-related aversive memory behaviors. On day 10, there was no significant difference in the CPA scores among all groups, which indicated that the pain-related aversive memory behaviors had disappeared with pain relief. On day 12, there was no significant difference in the CPA score between the Carr + NS group and NS + NS group. Compared with that of the Carr + NS group, the CPA score in the Carr + Carr group was again significantly higher (*P* < 0.05), indicating that the second injection of Carr had retrieved the pain-related aversive memory behaviors in the second group (Fig. 1C and D). Then, we examined the number of c-Fos-positive cells in the IC by IF. Compared with that in the Carr + NS group, the number of c-Fos–positive cells in the IC in the Carr + Carr group was significantly increased (*P* < 0.001; Fig. 1E and F). The above results indicate that the 2 crossover Carr injections successfully established the pain memory model and that pain memory could induce pain-related aversive memory behaviors, which might be related to the IC.

### IC lesions can prevent relapse of pain-related aversive memory behaviors

**Fig. 2:**
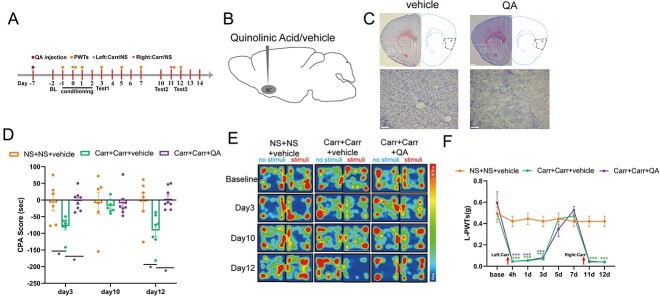
IC lesioning blocks pain-related aversive memory behaviors. A) Timeline for Carr injections and CPA testing. B) Schematic of drug injection (quinolinic acid/vehicle). C) Representative images of IC lesions (scale bars: 200/50 μm). D) Aversive memory behaviors following IC lesioning during the CPA test (2-way ANOVA, ^*^P < 0.05, *n* = 6–8 mice). E) Heatmap of each group at different times. F) L-PWTs hindpaw in each group (2-way ANOVA, ^*^^*^^*^P < 0.001, ^*^^*^^*^compared with the NS + NS + vehicle group; ^###^*P* < 0.001, ^###^compared with the Carr + Carr + vehicle group. *N* = 7 mice).

To investigate whether the IC is involved in pain-related aversive memory behaviors, we injected quinolinic acid into the IC in the Carr + Carr + QA group, whereas the NS + NS + Vehicle group and Carr + Carr + Vehicle group were injected with 0.9% saline into the IC (Fig. 2A–C). The CPA results indicated that on day 3, the CPA score of the Carr + Carr + Vehicle group was significantly higher than that of the NS + NS + Vehicle group (*P* < 0.05) and that of the Carr + Carr + QA group (*P* < 0.05), suggesting that drug-induced damage to the IC blocked the formation of pain-related aversive memory behaviors in the model mice. On day 10, the CPA scores of the mice in each group were not significantly different, indicating that the pain-related aversive memory behaviors disappeared with pain relief. On day 12, the CPA score in the Carr + Carr + Vehicle group was significantly higher than that in the NS + NS + Vehicle group (*P* < 0.05) and that in the Carr + Carr + QA group (*P* < 0.05; Fig. 2D and E). The results suggest that IC lesioning can block the relapse of pain-related aversive memory behaviors in model mice. From the L-PWT results, we observed that the L-PWTs of the Carr + Carr + Vehicle group and Carr + Carr + QA group both decreased when Carr was injected relative to those of the NS + NS + Vehicle group (*P* < 0.001; Fig. 2F). The above results show that the IC is involved in pain-related aversive memory behaviors, not pain sensation.

### EA effectively blocks pain-related aversive memory behaviors induced by 2 crossover injections of Carr

**Fig. 3:**
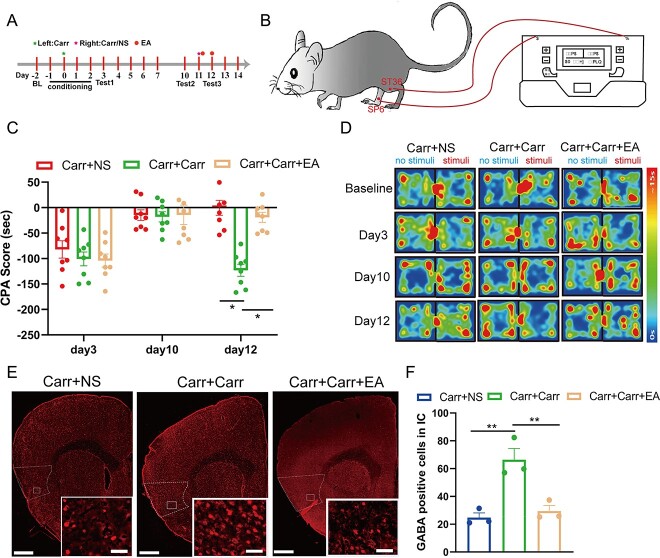
EA effectively blocks the pain-related aversive memory behaviors induced by 2 crossover injections of Carr in model mice. A) Experimental timeline for the CPA test and EA treatment. B) Schematic of EA treatment on the bilateral ST36 and SP6. C) Aversion-like behavioral effects of the EA stimulus in model mice (2-way ANOVA, ^*^P < 0.05, *n* = 7/8 mice). D) Heatmaps of the trajectory chart of each group in the CPA tests. (E) GABA-positive neurons in the IC in each group (scale bars: 200/20 μm). F) Numbers of GABA-positive neurons in the IC (1-way ANOVA, ^*^^*^^*^P < 0.001, *n* = 3 mice per group).

The timeline of the experimental design is shown in Fig. 3A. EA plays an important role in mitigating pain-related emotional disorders; therefore, we next investigated whether EA could regulate pain-related aversive memory behaviors in model mice. The CPA scores on day 3 after the first Carr injection were similar among the groups (*P* > 0.05). On day 10, the CPA scores of each group were again not significantly different, indicating that the pain-related aversive memory behaviors disappeared with pain relief. On day 12, the CPA score of the Carr + Carr group was significantly higher than that of the Carr + NS group (*P* < 0.05) and that of the Carr + Carr + EA group (*P* < 0.05; Fig. 3C and D). These results suggest that EA blocked the relapse of pain-related aversive memory behaviors in model mice. Then, we counted the number of GABA-positive neurons in the IC. The IF results showed that the model group contained significantly greater numbers of GABA-positive neurons than the control group (*P* < 0.05; Fig. 3E and F). These results indicate that GABAergic neurons may be associated with pain-related aversive memory behaviors.

### GABAergic neurons in the IC are involved in pain-related aversive memory behaviors induced by 2 crossover injections of Carr

**Fig. 4:**
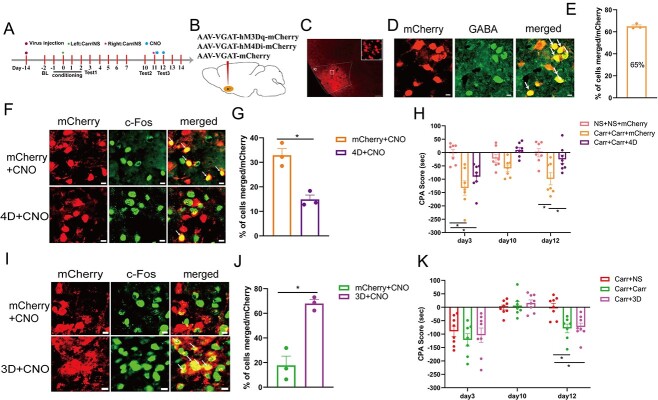
Activity changes in GABAergic neurons in the bilateral IC modulate the relapse of pain-related aversive memory behaviors induced by 2 crossover injections of Carr. A) Flow chart of the experimental design. B) Schematic of viral injection (AAV2/9-VGAT1-hM3Dq/hM4Di-mCherry-WPRE-pA or AAV2/9-VGAT1-mCherry-WPRE-pA). C) Viral expression in the IC after injection (scale bar: 200/10 μm). D) GABAergic neuron markers are colocalized with mCherry in the IC (scale bars: 20 μm). E) Percentage of mCherry and GABAergic neuron colocalization (*n* = 3 mice). F, I) Typical images of mCherry (red) and c-Fos (green) expression in the IC (scale bars: 20 μm, *n* = 3 mice). G) A significantly lower proportion of colocalization between mCherry (red) and c-Fos (green) was observed in the 4D + CNO group than in the mCherry+CNO group (*t*-test, ^*^P < 0.05, *n* = 3 mice). H, K) Aversive memory behavioral effects following chemogenetic inhibition or chemogenetic activation of IC^GABA^ neurons in pain-memory mice: CPA scores in each group (2-way ANOVA, ^*^P < 0.05; *n* = 7/8 mice). J) A significantly higher proportion of colocalization between mCherry (red) and c-Fos (green) was observed in the 3D + CNO group than in the mCherry + CNO group (*t*-test, ^*^P < 0.05, *n* = 3 mice).

To verify the role of GABAergic neuron activity in the IC in the relapse of pain-related aversive memory behaviors, we used a Cre-dependent viral vector approach to express Gi/Gq-coupled DREADDs selectively in IC GABAergic neurons and examined the consequences of decreasing neuron activity on pain-related aversive memory behaviors induced by 2 crossover injections of Carr. We infused an adeno-associated virus (AAV2/9-VGAT1-hM3Dq/hM4Di-mCherry-WPRE-pA) into the IC on day −14 (Fig. 4A and B). Activation of the receptor hM4Di by i.p. injection of CNO (2 mg/kg) led to a decrease in the colocalization of c-Fos and mCherry (Fig. 4F and G), and activation of hM3Dq led to an increase in colocalization (Fig. 4I and J). These results indicated that we could modulate the activation of GABAergic neurons in the IC by a chemogenetic method. Then, 2 weeks after virus infusion, we followed the pain memory model and CPA test, which induced pain-related aversive memory behavior (Fig. 4A and H). To confirm the role of these neurons in the IC, all mice were injected i.p. with CNO (2 mg/kg) to specifically inhibit the activation of GABAergic neurons in the IC on days 11–12. The results of the CPA test showed that chemogenetic inhibition of GABAergic neurons by CNO on days 11–12 reversed the effect of the crossover injections of Carr, blocking the relapse of pain-related aversive memory behaviors (Fig. 4H). In contrast, when we specifically activated GABAergic neurons by a chemogenetic method on day 11 instead of injecting Carr, the mice in the Carr + 3D group showed aversive memory behavior. In other words, the activation of GABAergic neurons played a similar role to the second injection of Carr in inducing pain-related aversive memory behavior relapse (Fig. 4K). Taken together, these results indicate that GABAergic neurons in the IC can bilaterally modulate the pain-related aversive memory behaviors induced by 2 crossover injections of Carr.

### KOR in IC participates in the processing of pain-related aversive memory behaviors induced by 2 crossover injections of Carr

**Fig. 5:**
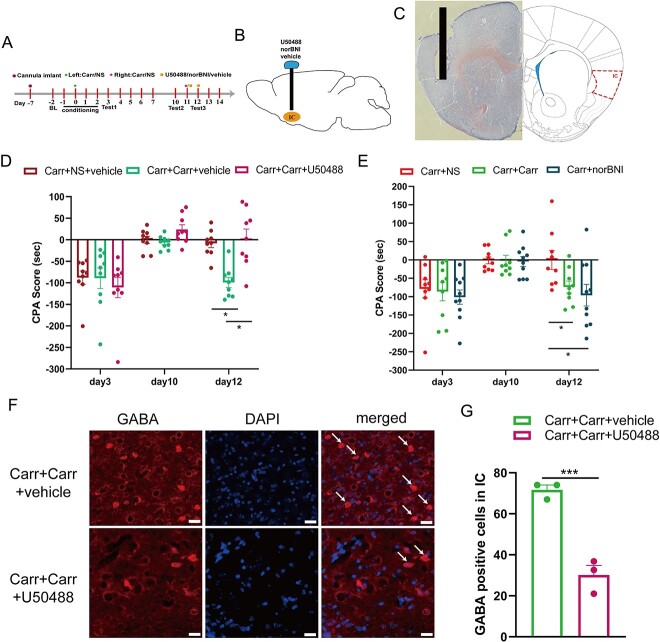
Changing the activation of KOR in the IC bilaterally regulates the relapse of aversive memory behaviors induced by crossover injection of Carr. A) Flow chart of the experimental design. B) Schematic of drug delivery (U50488/norBNI/vehicle). C) Representative diagram of the IC after cannula implantation (scale bars: 200 μm). D, E) Aversion-like behavioral effects following KOR agonist and antagonist application in different groups (2-way ANOVA, ^*^P < 0.05, *n* = 9 mice). F) GABAergic neurons in the right IC of Carr + Carr + vehicle and Carr + Carr + U50488 mice (scale bar: 20 μm). G) Number of GABA-positive neurons in the right IC according to IF (*t*-test, ^*^^*^^*^P < 0.001, *n* = 3 mice per group).

Previous studies have shown that KOR is widely distributed in the IC (Pina et al. 2020). To further verify that KOR is necessary and sufficient for pain-related aversive memory behaviors, we implanted a cannula into the bilateral insular cortices 1 week before the first Carr injection (Fig. 5A). A diagram of the cannulation location and a schematic representation of the injection of the cannula tip into the IC in the mouse brain are shown in Fig. 5B and C. Next, we determined whether an increase in KOR activation in the IC might be involved in pain-related aversive memory behaviors by examining the effects of local administration of the KOR agonist U50488 into the IC on days 11–12. We found that pretreatment with U50488 (10 μg/μL/side) on days 11–12 blocked the pain-related aversive memory behaviors induced by 2 crossover injections of Carr in the CPA test (*P* < 0.05; Fig. 5D). Then, we decreased KOR activity in the IC through local administration of the KOR antagonist norBNI (5 μg/μL/side) into the IC on days 11–12. We measured aversive memory behaviors by the CPA test and found that inhibiting KOR activity appeared to play a similar role to the 2 crossover injections of Carr in inducing the relapse of pain-related aversive memory behaviors (*P* < 0.05; Fig. 5E). Then, we used IF to detect the expression of GABAergic neurons in the IC after U50488 injections. Compared with that of the Carr + Carr + Vehicle group, the number of GABAergic neurons in the IC of the Carr + Carr + U50488 group was significantly decreased (*P* < 0.001; Fig. 5F and G), further supporting the idea that KOR is crucially involved in the relapse of pain-related aversive memory behaviors induced by 2 crossover injections of Carr.

### Activation of GABAergic neurons reversed the effect of KOR in blocking the relapse of pain-related aversive memory behaviors

**Fig. 6:**
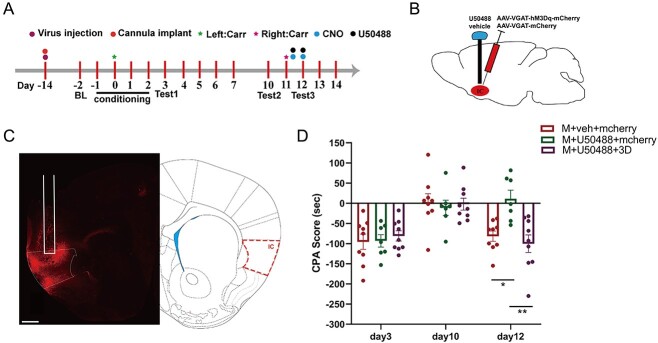
Activation of GABAergic neurons in the IC reversed the effects of KOR agonists in blocking the relapse of pain-related aversive memory behaviors. A) Flow chart of the experimental design. B) Schematic of drug delivery and virus injection (U50488 or vehicle and AAV2/9-VGAT1-hM3Dq-mCherry-WPRE-pA or AAV2/9-VGAT1-mCherry-WPRE-pA, respectively). C) Representative images of the IC after cannula implantation and virus injection (scale bar: 200 μm). D) Aversive memory behaviors following KOR activation via agonist administration and chemogenetic activation of GABAergic neurons in the IC in different groups (2-way ANOVA, ^*^  *P* < 0.05, ^*^^*^P < 0.01, *n* = 9 mice).

KOR has a regulatory effect on GABAergic neurons in the IC. To investigate whether KOR in the IC can regulate pain-related aversive memory behaviors by activating GABAergic neurons, we conducted the following experiments (Fig. 6A). First, on day 14, we infused an adeno-associated virus (AAV2/9-VGAT1-hM3Dq -mCherry-WPRE-pA or AAV2/9-VGAT1-mCherry-WPRE-pA) into the IC, and then we implanted a cannula in the bilateral IC (Fig. 6B and C). Next, we injected Carr on days 0 and 11 and conducted the CPA test on certain days. On days 11–12, all mice were first administered U50488 via injection into the IC; 10 min later, they were intraperitoneally injected with CNO (2 mg/kg) to specifically activate the GABAergic neurons in the IC. The CPA results showed that KOR prevented the relapse of pain-related aversive memory behaviors; however, subsequent activation of GABAergic neurons in the IC reversed the effect of KOR activation on day 12 (*P* < 0.01; Fig. 6D). These findings indicate that GABAergic neuron-dependent activation of KOR in the IC is crucially involved in the relapse of pain-related aversive memory behaviors induced by 2 crossover injections of Carr.

### Inhibition of KOR or activation of GABAergic neurons in the IC effectively attenuated the effect of EA on regulating aversive memory behaviors

**Fig. 7:**
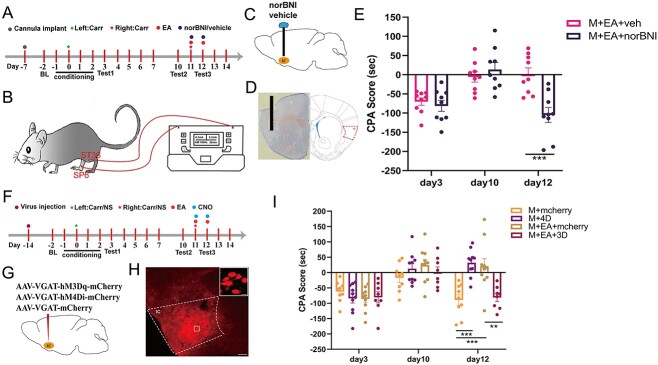
Pharmacological blockade of KOR or chemogenetic activation of GABAergic neurons in the IC effectively attenuates the EA effect. A, F) Flow chart of the experimental design. B) Schematic of EA treatment on bilateral ST36 and SP6. C) Schematic of drug delivery (norBNI/vehicle). D) Sample image of the IC after cannula implantation. E) Aversion-like behavioral effects of EA treatment and KOR antagonists in different groups (2-way ANOVA, ^*^^*^^*^P < 0.001, *n* = 9 mice). G) Schematic of viral injection (AAV2/9-VGAT1-hM3Dq/hM4Di-mCherry-WPRE-pA or AAV2/9-VGAT1-mCherry-WPRE-pA) in the IC. H) Expression of virus in the IC after viral injection (scale bar: 100/10 μm). I) Aversive memory behavioral effects of the chemogenetic inhibition/activation of IC^GABA^ neurons combined with EA treatment in different groups (2-way ANOVA, ^*^^*^P < 0.01, ^*^^*^^*^P < 0.001, *n* = 9 mice).

The results of the above experiment showed that KOR and GABAergic neurons in the IC are crucial to the regulation of pain-related aversive memory behaviors induced by 2 crossover injections of Carr. Furthermore, we previously verified the effect of EA on pain-related aversive memory behaviors. Therefore, we inhibited KOR in the IC and activated GABAergic neurons to determine whether these changes could reverse the effect he function of EA treatment. We first implanted a cannula into the bilateral IC on day 7 (Fig. 7A–D). The 2 groups were subjected to the CPA test. Compared with the M + EA + veh group, the M + EA + norBNI group had lower CPA scores on day 12 (*P* < 0.001; Fig. 7E). Next, we injected the adeno-associated virus into the IC (Fig. 7F–H), and the 4 groups were subjected to the CPA test again. Compared with the M + mCherry group, the M + 4D group and M + EA + mCherry group had higher CPA scores (*P* < 0.001). Compared with the M + EA + mCherry group, the M + EA + 3D group had lower CPA scores (*P* < 0.01; Fig. 7I). The above results show that pharmacological inhibition of KOR activity or chemogenetic activation of GABAergic neurons in the IC powerfully reversed the effect of EA on blocking pain-related aversive memory behaviors induced by 2 crossover injections of Carr.

## Discussion

IC has been thoroughly demonstrated to play a key role in regulating negative emotions such as aversion (Gehrlach et al. 2019). However, the potential mechanism by which the neurons or receptors in the IC affect pain-related aversive memory behavior relapse and the related mechanism of action of EA in these behaviors are poorly understood. Our study is the first to reveal how the activity changes in GABAergic neurons or KOR in the IC regulate the pain memory-related aversive memory behaviors induced by 2 crossover injections of Carr. Then, we studied whether the mechanism by which EA blocks pain-related aversive memory behaviors is related to the activity of GABAergic neurons or KOR in the IC. Here, we found that chemogenetic inhibition of GABAergic neurons or pharmacological agonism of KOR in the IC blocked pain-related aversive memory behaviors. Conversely, specific activation of GABAergic neurons or antagonism of KOR in the IC plays a similar role as 2 crossover injections of Carr in inducing the relapse of pain-related aversive memory behaviors. Additionally, the chemogenetic activation of GABAergic neurons in the IC reversed the effect of KOR agonists in blocking pain-related aversive memory behaviors. EA also blocked pain-related aversive memory behaviors in a manner related to the activity of KOR and GABAergic neurons in the IC.

Patients with chronic pain form persistent pain memories and, under identical circumstances, show avoidance behaviors. Pain-related aversive memory behaviors indelibly influence subsequent perception, which is currently considered the key reason why chronic pain is difficult to cure (Chen et al. 2000). First, we established a pain memory model with crossover injections of 0.5% Carr, consistent with previous studies on this model (Kissin et al. 2006). Then, we observed that aversive memory behaviors were formed following the first injection of Carr in the left hind paw and were retrieved following a second injection of Carr in the right hind paw. The results of numerous clinical studies concur with our findings; for example, children who had a negative evaluation of pain memories showed higher increases in pain ratings than children who had a positive estimation of pain memories (Noel et al. 2012). Pain-related aversive memory severely reduces the patient's subsequent recovery and quality of life.

The IC plays a crucial role in aversion and memory; a previous study demonstrated, for example, that the formation and storage of taste aversion memory is closely associated with plastic changes in the IC (Guzman-Ramos and Bermudez-Rattoni 2012). When GABAergic synaptic transmission in the IC is decreased, conditioned taste aversion memory in mice is impaired (Toyoda et al. 2020). Recent research in mice has also shown that the activity of VIP^+^ interneurons in the aIC is required for the retrieval of aversive memory behavior following implementation of a fear conditioning paradigm (Ramos-Prats et al. 2022). Moreover, it has been demonstrated that using optogenetic techniques to inhibit the posterior insula cortex (pIC) during footshock impaired threat memory and reduced aversive somatosensory information such as acute fear behavior in mice (Berret et al. 2019), suggesting that the pIC can detect aversive internal states to make behavioral strategies (Gehrlach et al. 2019). The above studies illustrate that IC is closely related to aversive memory behaviors. Our study found that lesioning the IC blocked the relapse of pain-related aversive memory behaviors induced by 2 crossover injections of Carr, but it did not affect the nociceptive sensation. The reason for this phenomenon might lie in the different functions of different regions in IC. According to previous reports, IC can be divided into aIC and pIC (Ramos-Prats et al. 2022). The aIC is connected with pain-related emotional feelings (Chen et al. 2018; Beauchaine et al. 2019), and the pIC is related to pain sensation (Meier et al. 2018). Furthermore, in our study, the model mice showed significant differences in the numbers of c-Fos-positive cells and GABA-positive cells in the IC, which is consistent with our behavioral results.

It has been reported that the activity of GABAergic neurons (Dehkordi et al. 2018; Sun et al. 2020b) and KOR (Margolis and  Karkhanis 2019; Zan et al. 2021) regulates aversive behaviors. However, little is known about how GABAergic neurons and KOR in the IC modulate the pain-related aversive memory behaviors induced by 2 crossover injections of Carr. Consequently, we used a chemogenetic method to control the activity of GABAergic neurons in the IC and a pharmacological method to regulate KOR activity in the IC. We found that both methods had an effect on pain-related aversive memory behaviors induced by 2 crossover injections of Carr. A previous study demonstrated that activation of KOR in the IC directly inhibited the activity of layer 5 GABAergic neurons in the IC (Pina et al. 2020). Our results showed that U50488 (a KOR agonist) significantly decreased the number of GABA-positive neurons in the IC with respect to the vehicle group, confirming the findings of previous research.

EA is widely applied in treating pain (Zhang et al. 2014; Kelly and Willis 2019; Jiang et al. 2021) and emotional disorders (Amorim et al. 2018; Smith et al. 2018) because of its effectiveness and lack of side effects. Many studies have reported that EA stimulation at the Zusanli (ST36) and Sanyinjiao (SP6) acupoints can reduce mechanical pain sensitivity and anxiety-like behaviors in SNI mice (Wu et al. 2022). Kong et al.’s (2002) study demonstrated that EA caused fMRI signal increases in some brain regions, such as the insula. Additionally, 100 Hz EA at ST36 and SP6 has been shown to suppress cocaine-induced conditioned place preference via KOR in the nucleus accumbens (Hou 2016). In our experiment, we applied 100 Hz EA at the bilateral ST36 and SP6 for 30 min on days 11–12 to observe the effect of EA on pain-related aversive behavior memory behaviors. We found that EA treatment blocked the relapse of pain-related aversive memory behaviors in model mice. Furthermore, we found that the activity of GABAergic neurons and KOR in the IC was involved in pain-related aversive memory behaviors. Our results showed that EA can block pain-related aversive memory behaviors by activating the KOR, further inhibiting GABAergic neurons in the IC.

Overall, the present study demonstrated a new mechanism by which GABAergic neurons and KOR in the IC regulate pain-related aversive behavior memory induced by 2 crossover injections of Carr, ultimately revealing the mechanism by which EA blocks the relapse of pain-related aversive memory behaviors.

## Author contributions

Siqi Xiao (Conceptualization, Data curation, Formal analysis, Supervision, Validation, Writing—original draft, Writing—review & editing), Haiju Sun (Conceptualization, Formal analysis, Investigation, Validation, Writing—original draft), Yichen Zhu (Data curation, Formal analysis, Investigation, Validation, Writing—original draft), Zui Shen (Conceptualization, Data curation, Formal analysis, Validation), Xixiao Zhu (Data curation, Formal analysis, Investigation, Validation), Ping-an Yao (Data curation, Formal analysis, Investigation), Yifang Wang (Data curation, Investigation, Validation), Chi Zhang (Investigation, Supervision, Validation), Wei Yu (Data curation, Investigation), Zemin Wu (Investigation, Supervision, Validation), Jing Sun (Conceptualization, Investigation, Supervision, Writing—original draft), Chi Xu (Conceptualization, Investigation, Validation), Junying Du (Investigation, Supervision), Xiaofen He (Data curation, Investigation), Jianqiao Fang (Conceptualization, Data curation, Formal analysis, Investigation, Methodology, Resources, Supervision, Validation, Writing—original draft, Writing—review & editing), and Xiaomei Shao (Conceptualization, Data curation, Formal analysis, Funding acquisition, Investigation, Methodology, Resources, Supervision, Validation, Writing—original draft, Writing—review & editing)

## Funding

This study was supported by grants from the National Natural Science Foundation of China (82074518) and the Natural Science Foundation of Zhejiang Province (LY21H270010). The animal study was reviewed and approved by the Animal Ethics Committee of Zhejiang Chinese Medical University (ZSLL, 2017-183). The content is solely the responsibility of the authors and does not necessarily represent the official views of our sponsors.

*Conflict of interest statement*: None declared.

## Data availability

Data are available upon request.

## Supplementary Material

Supplement_1_bhad321
