# Supplementary material for: Electroacupuncture alleviates the relapse of pain-related aversive memory by activating KOR and inhibiting GABAergic neurons in the insular cortex
Source: Cereb Cortex. 2023 Sep 7;33(20):10711–21. doi: 10.1093/cercor/bhad321 (PMC10560575; doi:10.1093/cercor/bhad321)
Supplement: Supplement_1_bhad321 [file supplement_1_bhad321.docx]

**Supplement 1**

**EA alleviates pain-related aversive behavior induced by injection of KOR antagonist norBIN**

We added the design to confirm the effect of EA on pain-related memory behavior as below (Supplementary fig 1). First, we implanted cannuclas into the bilateral insular cortices one week before the Carr injection. Then, we decreased KOR activity in the IC through local administration of the KOR antagonist norBNI into the IC on days 11-12. The EA intervention was initiated 5 minutes after the norBNI injection on days 11-12. (Supplementary Figure 1 A-C) The aversive memory behaviors were measured by the CPA tests as previously described. As the results showed, mice performed pain related aversive behavior on day 3 and returned to normal level on day 10. Consistent with previous experiments, mice received 2-day’s norBNI microinjection showed the aversive behavior again. Interestingly, the aversive behaviors were significantly released by the EA intervention followed the norBNI microinjection (Supplementary Figure 1 D). The results suggested that EA could prevent the pain-related memory behavior, aversive, directly.

Summary, we concluded that EA could alleviate pain-related memory behavior directly. Certainly, we have no evidence to exclude the effect of EA on pain-related memory behavior by inhibit pain behavior currently.

**
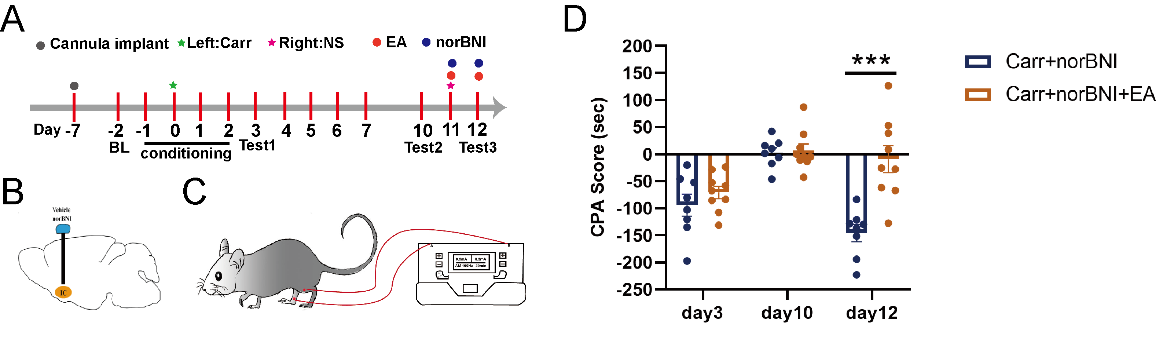
**

**Supplementary fig 1 EA alleviates pain-related aversive behavior induced by injection of KOR antagonist norBIN.** (A) Experimental timeline for the CPA test and EA treatment. (B) Schematic of drug delivery (norBNI). (C) Schematic of EA treatment on the bilateral ST36 and SP6. (D) Aversion-like behavioral effects of the EA stimulus in model mice (two-way ANOVA, ****P* < 0.001, n=8 mice).
